# Actin’ up: Herpesvirus Interactions with Rho GTPase Signaling

**DOI:** 10.3390/v3040278

**Published:** 2011-03-24

**Authors:** Céline Van den Broeke, Herman W. Favoreel

**Affiliations:** Department of Virology, Parasitology and Immunology, Faculty of Veterinary Medicine, Ghent University, Sint-Pietersnieuwstraat 25, B-9000 Ghent, Belgium; E-Mail: herman.favoreel@ugent.be

**Keywords:** herpesvirus, Rho GTPases, actin, cytoskeleton

## Abstract

Herpesviruses constitute a very large and diverse family of DNA viruses, which can generally be subdivided in alpha-, beta- and gammaherpesvirus subfamilies. Increasing evidence indicates that many herpesviruses interact with cytoskeleton-regulating Rho GTPase signaling pathways during different phases of their replication cycle. Because of the large differences between herpesvirus subfamilies, the molecular mechanisms and specific consequences of individual herpesvirus interactions with Rho GTPase signaling may differ. However, some evolutionary distinct but similar general effects on Rho GTPase signaling and the cytoskeleton have also been reported. Examples of these include Rho GTPase-mediated nuclear translocation of virus during entry in a host cell and Rho GTPase-mediated viral cell-to-cell spread during later stages of infection. The current review gives an overview of both general and individual interactions of herpesviruses with Rho GTPase signaling.

## 1. Introduction

Structural integrity of a cell, intracellular organization and transport, cell shape and motility, cell division—all these, and many other central aspects of cellular life critically rely on a functional cytoskeleton. At the same time, the cytoskeleton constitutes both a formidable barrier and a powerful tool for viruses trying to invade and take over a cell. It is therefore not surprising that herpesviruses have evolved several interactions with the cytoskeleton to facilitate their replication and spread. Increasing evidence indicates that, at multiple stages of their replication cycle, herpesviruses interfere with cellular signaling pathways that affect the structure and organization of the cytoskeleton. The current review will discuss recent progress in our understanding of how herpesviruses interfere with signaling pathways that affect the actin cytoskeleton.

Cytoskeletal signaling is mainly initiated by extracellular stimuli that activate cell surface receptors such as G protein coupled receptors (GPCR), integrins and receptor tyrosine kinases (RTK). Signaling downstream of these receptors culminates in activation of specific Rho GTPases [1–3]. Rho GTPases constitute a family of small G proteins that are critically involved in a variety of vital cell functions such as cell proliferation, apoptosis, and gene expression and are considered to be the main regulators of the actin cytoskeleton. They act as molecular switches that cycle between an active GTP-bound form and an inactive GDP-bound form. This ‘switch’ is regulated by three sets of proteins: guanine nucleotide-exchange factors (GEFs) that catalyze the exchange of GDP for GTP [4], GTPase activating proteins (GAPs) that stimulate the intrinsic GTPase activity of the Rho GTPases [5] and guanine nucleotide-dissociation inhibitors (GDIs), whose main role appears to be to prevent spontaneous activation of Rho GTPases [6]. In their active, GTP-bound state, Rho GTPases perform their regulatory function through a conformation-specific interaction with target effector proteins. Although 20 different Rho GTPases have been identified in mammals, attention has focused predominantly on the three most common members of the family: RhoA, Rac1, and Cdc42. The activation of RhoA, Rac1 or Cdc42 leads to the assembly of contractile actin-myosin filaments (stress fibers), protrusive actin-rich lamellipodia and protrusive actin-rich filopodia, respectively [7–11]. The signaling pathways downstream of RhoA, Rac1 and Cdc42 are interwoven and the RhoA pathway generally counteracts the Rac1 and Cdc42 pathways [12–14]. Despite the differences in actin structures induced by each of these three Rho GTPases, activation of either leads to actin nucleation and polymerization, and influences actomyosin dynamics. A common series of signal transduction pathways controlled by each GTPase leads to both the formation (actin polymerization) and the organization (filament bundling) of actin filaments. For example, downstream signaling of RhoA, Rac1 or Cdc42 leads to stabilization of F-actin through phosphorylation of LIM kinase (LIMK). Specific downstream targets of Cdc42/Rac1/RhoA and timing and location of their activation define which type of F-actin structure will be formed.

The current review aims at giving a comprehensive overview of the known interactions between herpesviruses and Rho GTPase signaling, rather than giving an overview on the variety of interactions between these viruses and the actin cytoskeleton. As a consequence, different effects of herpesviruses with the cytoskeleton that have not been related to effects on Rho GTPase signaling will not be discussed. These have been reviewed extensively elsewhere [15,16].

## 2. Entry

The past years have revealed that herpesvirus entry is often, if not generally, associated with Rho GTPase signaling. This is particularly evident for alpha- and gammaherpesviruses. Less information is available for betaherpesviruses. Herpesviruses gain access to the cytosol of a host cell by fusion of the envelope with a host membrane. Depending on the virus and the type of host cell, this fusion event may occur directly at the cell surface or upon uptake of the virus via endocytosis, macropinocytosis, or phagocytosis. The need and specificity of Rho GTPase signaling and actin rearrangements during herpesvirus entry may very well depend on which of these uptake routes are engaged during infection of a particular cell. Indeed, direct fusion at the cell surface on itself may not require dramatic actin rearrangements. However, this entry route confronts the viral particle with the cortical actin barrier just beneath the plasma membrane, which likely needs to be resolved to some extent by a viral triggered mechanism. Engaging one of the endocytic uptake routes may circumvent this problem, but may still rely on virus-induced Rho GTPase signaling during the initial interaction of the virus with the cell since several of these uptake routes, particularly macropinocytosis and phagocytosis, require Rho GTPase signaling and significant actin rearrangements to proceed [17,18]. The consequences of triggering Rho GTPase signaling during the initial interactions of herpesviruses with host cells may stretch well beyond successful delivery of viral particles in the cytoplasm, and may affect virus transport to the nucleus, efficiency of viral replication, and even egress of progeny virus.

### 2.1. Alphaherpesviruses

**Figure 1.**
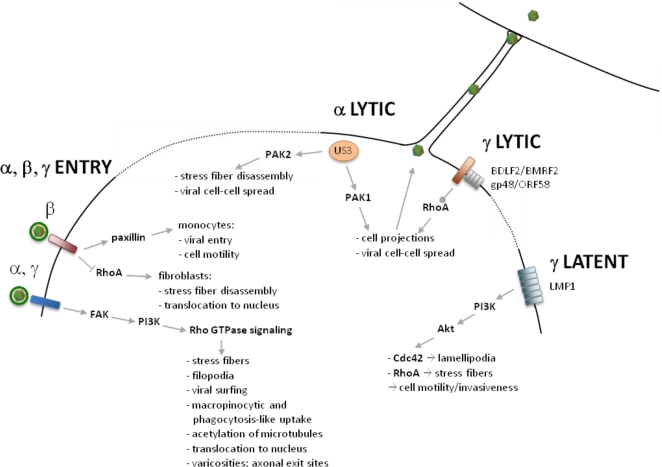
Overview of herpesvirus interactions with Rho GTPase signaling during virus entry, viral lytic infection and viral latent infection.

Several lines of evidence suggest that alphaherpesviruses interfere at different points with Rho GTPase signaling pathways during viral entry (Figure 1). Alphaherpesvirus entry involves fusion of the viral envelope with the host cell membrane which may occur at the plasma membrane or upon endocytic or phagocytic uptake of the virus [19–21].

Regardless of the viral entry pathway, alphaherpesviruses first contact the cell by interaction of viral envelope proteins gB and gC with heparan sulfate moieties on the cell surface. Live cell imaging of HSV-1 infected retinal pigment epithelial cells demonstrated that many virions first attach to filopodia-like structures and move unidirectionally along these filopodia to reach the cell body [22]. HSV not only travels along filopodia during entry, but may also actively induce filopodia formation at this stage through activation of Cdc42 signaling [21]. Recently, inhibitor studies showed that phosphoinositide 3 kinase (PI3K) is important for HSV-induced filopodia formation, RhoA activation, and subsequent viral entry [23].

Viral induction of filopodia and viral surfing along filopodia likely aid in recruiting high numbers of virus particles towards entry receptors on the cell surface, facilitating subsequent virus entry. Although not formally shown as of yet, it is possible that HSV surfing along filopodia occurs through myosin/actin-dependent retrograde flow, as described for retroviruses [24]. Perhaps in support of this, non-muscle myosin IIA has recently been reported to appear on the cell surface during HSV entry and to serve as a receptor for the gB envelope protein of HSV [25]. In support of a role for gB in viral surfing, an HSV recombinant lacking gB was impaired in filopodia binding, and recombinant gB by itself demonstrated some ability to surf along filopodia [26]. Although gB is required for viral surfing along filopodia, this envelope protein does not appear to be involved in the Rho GTPase-mediated induction of filopodia during HSV entry.

Another alphaherpesvirus envelope protein, gD, has been associated with Rho GTPase signaling during viral entry [21,27]. The gD envelope protein can engage different classes of cellular receptors during viral entry, including nectins. Nectins are cell adhesion molecules that are able to activate Rho GTPase signaling, including Cdc42 and Rac1 signaling, which may lead to formation of filopodia and lamellipodia [28,29]. Chinese hamster ovary (CHO) cells are not susceptible to infection by HSV unless a heterologous gD receptor, which is normally lacking in these cells, is expressed. In nectin-1-expressing CHO cells, HSV entry was associated with nectin-1-dependent activation of Cdc42, which in turn was associated with the formation of filopodia [21]. In this cell type, nectin-1 also activated RhoA signaling which was associated with more prominent stress fibers. Nectin-1-mediated Rho GTPase signaling and cytoskeletal changes culminated in the efficient uptake of the virus through a previously uncharacterized phagocytic route [21]. In further support of an important role for gD/nectin-1 signaling in filopodia formation during HSV entry, antibodies directed against nectin-1 were able to inhibit filopodia formation and subsequent HSV entry in retina pigment epithelial cells [22].

Rho GTPase signaling through gD/nectin-1 has also been reported for a swine alphaherpesvirus, pseudorabies virus (PRV) during viral entry in primary trigeminal ganglion neurons, a central target cell type for different alphaherpesviruses [27]. PRV entry in these cells resulted in Cdc42- and Rac1-mediated formation of pre-synaptic boutons or varicosities along the axons of the TG neurons. Experiments with gD-deleted PRV, recombinant gD, and nectin-1 antibodies showed that varicosity formation is driven by the interaction of the envelope protein gD with nectin-1. In line with these findings is the observation that interaction of HSV-1 gD with nectin-1 in mouse hippocampus neurons results in a substantial increase in the number of varicosities [29]. Varicosities were found to serve as axonal exit sites for progeny PRV virions [27]. This is consistent with other reports where axonal egress of alphaherpesviruses, including HSV-1, was observed at varicosities in neurons [30,31].

Although it is obvious that alphaherpesvirus entry in host cells is associated with Rho GTPase signaling, considerable variation has been reported regarding the Rho GTPase members involved. This variation appears to mainly depend on the cell type investigated. Brief Cdc42 activation and sustained RhoA activation occurs during HSV-1 infection of CHO-nectin-1 cells [21]. In MDCKII cells on the other hand, HSV-1 entry is associated with activation of Cdc42 and Rac1 [32], which is in line with the Cdc42- and Rac1-dependent signaling during PRV entry in TG neurons [27]. In keratinocytes, HSV-1 infection was reported not to depend on pathways involving either Rac1 or Cdc42 [33]. A possible explanation for the discrepancy in Rho signaling seen during alphaherpesvirus entry in different cell types may perhaps be found in different entry pathways engaged by the virus in these different cells. Thus, careful dissection of viral entry pathways may be of crucial importance to improve our understanding of the cytoskeletal signaling induced upon infection.

In order for cell surface receptors to activate Rho GTPase signaling, different intermediate molecules may be involved. Focal adhesion kinases (FAK) and phophatidyl inositol-3 kinase (PI3K) are two prominent intermediate signaling molecules. In CHO-nectin-1 cells, PI3K activity is involved in trafficking of HSV-1 virus particles through the cytosol to the nucleus, but not in viral attachment or penetration [34]. In line with these findings, phosphorylation of FAK is not involved in binding, but is crucial for the delivery of viral capsids to the nuclear periphery upon HSV-2 infection [35]. This increased nuclear delivery of capsids may point to increased microtubule-mediated capsid transport. In this context, it may be important to note that, in analogy to the gammaherpesvirus KSHV, infection with the alphaherpesvirus equine herpesvirus-1 (EHV-1) induces acetylation of microtubules [36], which may enhance microtubule motor-dependent trafficking [37]. Although it was previously shown that ROCK, a downstream effector of RhoA, was critical for EHV-1 infection [38], EHV-1-induced acetylation of microtubules did not depend on the activation of ROCK [36]. Nevertheless, ROCK activation is of critical importance for intracellular trafficking of EHV-1 particles since ROCK inhibition significantly decreased the number of capsids accumulating at the nucleus [36].

### 2.2. Betaherpesviruses

In betaherpesvirus HCMV infection, EGFR serves as a receptor and αvβ3 integrin as a coreceptor. Upon HCMV infection in fibroblasts, viral glycoproteins gB and gH independently bind to EGFR and αvβ3 integrin, respectively, to initiate viral entry and signaling. EGFR activates PI3K, while αvβ3 integrin signals to Src, and their coordinated action results in further downstream signaling. Both signaling pathways are involved in the decreased total and activated RhoA levels observed upon HCMV infection in fibroblasts [39]. HCMV entry is also associated with reduced phosphorylation of cofilin, a downstream protein in RhoA signaling, which correlates with the dramatic decrease in the number of actin stress fibers. Both HCMV-induced RhoA downregulation and disruption of stress fibers are crucial for the nuclear translocation of HCMV [39]. In monocytes, however, EGFR is involved in HCMV entry in a PI3K-independent manner. Nevertheless, HCMV-induced activation of EGFR in monocytes also triggers PI3K-dependent upregulation of N-WASP, an actin nucleator. This results in increased monocyte motility [40,41]. Recently, HCMV-mediated integrin and EGFR signaling in monocytes were both found to upregulate paxillin, an important signaling adaptor molecule that modulates interactions between multiple proteins involved in signaling, cytoskeletal rearrangements, cellular motility and adhesion. The interaction of HCMV with integrins but not with EGFR induces paxillin phosphorylation and thus activation. Paxillin activation is essential for efficient viral entry into target monocytes and for HCMV-induced monocyte motility, which may contribute to systemic viral spread and hematogenous dissemination [42]. Contrary to these findings in monocytes, HCMV was found to suppress expression of paxillin in fibroblasts, and HCMV infection was associated with disruption of focal adhesions in this cell type [43]. This again underscores the importance of the host cell type in the nature and consequences of herpesvirus interactions with Rho GTPase signaling.

### 2.3. Gammaherpesviruses

One of the best-documented interactions of herpesviruses with cytoskeletal signaling is triggered by the interaction of the RGD motif of KSHV gB with its cellular entry receptor α3β1integrin. In fibroblasts, this binding induces integrin signaling which contributes to viral entry. KSHV-induced integrin signaling in fibroblasts results in phosphorylation of FAK, which leads to the activation and phosphorylation of Src tyrosine kinases and PI3K. This in turn results in the activation of Rho GTPase signaling pathways. Cdc42 was activated from five minutes post infection, followed by sustained RhoA and ezrin activation, resulting in cytoskeletal rearrangements, including stress fiber and filopodia formation [44–46]. Although the KSHV-induced cytoskeleton-controlling signaling pathways are of pivotal importance for efficient viral entry, experiments with inhibitors of actin dynamics showed that actin polymerization *per se* did not seem to affect viral binding, entry, nuclear transport or infection in fibroblasts [44]. However, another study indicated that the role for actin dynamics in KSHV entry is cell type dependent. In endothelial cells, KSHV entry was also associated with profound actin rearrangements, including loss of stress fibers, dissolution of cortical actin, and formation of ruffles, lamellipodia, and filopodia. In this cell type, KSHV-induced actin rearrangements strongly contributed to viral entry [47]. One explanation for this apparent discrepancy may lie in differences in KSHV uptake route in fibroblasts *versus* endothelial cells [48]. In endothelial cells, KSHV uptake occurs via an actin-dependent macropinocytic pathway whereas uptake in fibroblasts occurs through an actin-independent clathrin-mediated endocytosis pathway [45,48]. In line with this, a recent study demonstrated that KSHV interaction with endothelial cells induces rapid tyrosine phosphorylation of c-Cbl, an adaptor protein typically involved in negative and positive regulation of signaling through ubiquitinylation and assembly platform functions, respectively. Tyrosine phosphorylated c-Cbl was identified as an adaptor protein that interacts with PI3K and myosin IIA, thereby driving macropinocytic uptake of KSHV [49]. However, some controversy exists on the route of KSHV uptake in endothelial cells since another report identified a clathrin-mediated endocytic route as the predominant gate of viral entry in this cell type [47]. Nevertheless, also in the latter report, actin dynamics were crucial for efficient viral uptake. Hence, in endothelial cells but not in fibroblasts, the effects of KSHV-induced Rho GTPase signaling on actin dynamics are important for efficient virus uptake.

In fibroblasts, the role of KSHV-induced Rho GTPase activation on virus entry appears to rely on its subsequent effects on microtubules, another major component of the host cytoskeleton. KSHV-induced Rho GTPase signaling results in acetylation and thereby stabilization of microtubules. Microtubule stabilization facilitated delivery of viral DNA to the nucleus, likely by promoting dynein-mediated transport of virus from the periphery to the nucleus. The stabilization of microtubules was found to be mediated by one of the downstream effectors of RhoA, mDia2, a member of the formin family [50].

Src tyrosine kinases appear to be crucial in KSHV-induced RhoA activation. On the one hand, KSHV induces Src activation, which in turn leads to RhoA activation. On the other hand, RhoA activation of mDia2 results in activation of Src. This creates a positive feedback loop that results in sustained Src activation, which is important during viral entry [51]. Src tyrosine kinases predominantly localize to the cytoplasmic surface of cholesterol- and sphyngolipid-enriched microdomains of the plasma membrane, so-called lipid rafts [52]. In line with this, Raghu and colleagues showed that lipid rafts of endothelial cells are critically involved in post-binding and entry stages of KSHV infection since lipid raft disruption resulted in decreased activation of PI3K, RhoA and mDia2 and a reduced nuclear delivery of viral DNA [53].

Taken together, entry by members of the three herpesvirus subfamilies have been reported to be associated with altered Rho GTPase signaling. Activation of FAK and its downstream activation of RhoA seems to be a conserved characteristic of alpha- and gammaherpesviruses, whereas betaherpesvirus entry may be associated with RhoA suppression. In either case, modulation of RhoA signaling enhances trafficking of viral particles to the nucleus.

## 3. Latent and Acute Infection

Increasing evidence indicates that herpesviruses also trigger Rho GTPase signaling and consequent actin rearrangements later in infection. Again, like for entry, less is known concerning betaherpesvirus interaction with Rho GTPases compared to alpha- and gammaherpesviruses. It will therefore be interesting to determine whether this points to an actual difference in ability of these viruses to interact with Rho GTPase signaling, or whether this particular field of research has been less explored thus far in betaherpesviruses. Both for alpha- and gammaherpesviruses, interactions with Rho GTPase signaling during later stages of infection are associated with the formation of cell projections and are thereby believed to promote viral spread. In addition, for gammaherpesviruses, interactions with Rho GTPase signaling during latency affect cell motility and invasion and may therefore be involved in malignancies associated with these viruses.

### 3.1. Alphaherpesviruses

It is becoming increasingly clear that lytic alphaherpesvirus infection is associated with alterations in Rho GTPase signaling and concomittant drastic rearrangements of the actin cytoskeleton. These rearrangements have been largely attributed to the viral US3 kinase, which is a conserved alphaherpesvirus serine/threonine kinase. For HSV-2, PRV, BHV-1 and MDV, the US3 kinase induces actin rearrangements consisting of the formation of long cellular projections and/or the disassembly of actin stress fibers [54–60]. US3-induced actin rearrangements are associated with increased intercellular virus spread. Virus can travel via the US3-induced cell projections to distant non-infected cells [56]. The observed actin rearrangements are a consequence of the interference of US3 with Rho GTPase signaling. A first indication that US3 affects Rho GTPase signaling came from studies by Murata *et al.* (2000). Based on experiments using different dominant active and negative constructs, it was hypothesized that US3 may regulate its effects on the actin cytoskeleton through Cdc42/Rac1 signaling pathways [58]. More recent studies on PRV showed that US3-mediated actin rearrangements depend on group A p21-activated kinases (PAKs), critical downstream effectors of the Cdc42/Rac1 signaling pathways [61]. Activation of group A PAKs induces actin rearrangements that are very similar to those observed upon US3 expression [62]. PRV US3 is able to phosphorylate and thereby activate both PAK1 and PAK2. Experiments using PAK knockout cell lines showed that the presence of PAK2 is critical for US3-mediated disassembly of actin stress fibers whereas PAK1 is important for efficient US3-mediated formation of cell projections [61].

Although the conserved phenotype of US3-mediated actin rearrangements suggests a conserved mechanism of action for different alphaherpesviruses, there are indications for differences in the mode of action of US3 of different alphaherpesviruses. Indeed, for PRV, HSV-2, and BHV-1, point mutations in conserved kinase regions of US3 showed that the catalytic kinase activity of US3 is crucial for reorganization of the actin cytoskeleton, while this was not the case for MDV [55,57,60,63,64]. Combined with the observation that the effect of MDV US3 on the actin cytoskeleton appears to be less dramatic (actin stress fiber disassembly but no reported cell projections), this suggests that US3 may also display non-catalytic mechanisms that affect actin stress fiber stability. Apart from US3-mediated actin rearrangements in the cytoplasm, lytic alphaherpesvirus infection is also associated with the formation of filamentous actin in the nucleus, although it is unknown if and how Rho GTPase signaling is involved in this process [65].

### 3.2. Gammaherpesviruses

The gammaherpesvirus Epstein-Barr virus (EBV) causes infectious mononucleosis and is associated with a number of malignancies. These cancers are characterized by the proliferation of EBV-infected cells and viral expression in these cells is limited to a subset of latent genes [66,67]. The LMP1 protein (latent membrane protein 1) is the major transforming protein and it is becoming increasingly clear that its expression activates signaling pathways leading to rearrangements of the actin cytoskeleton with implications for cell motility and invasiveness. Early reports showed that LMP1 expression leads to membrane ruffling and the formation of membrane protrusions [68,69]. In the late nineties and the early 2000s, more insights in these signaling pathways were obtained. LMP1-induced actin polymerization was found to depend on the induction of PI3K activity and subsequent phosphorylation and activation of Akt, a downstream target of PI3K involved in cell survival. Lamellipodia formation was mediated by the activation of Cdc42 through the transmembrane spanning regions of LMP1, while the CTAR1 region of LMP1 is crucial for Rho-mediated stress fiber formation [70,71].

A recent report demonstrates that LMP1-mediated actin signaling not only occurs via small Rho GTPases but also through activation of the protein kinase C (PKC) pathway. This leads to phosphorylation of ezrin, a member of the ezrin/moesin/radixin family that acts as a linker between the plasma membrane and the actin cytoskeleton. Phosphorylated ezrin translocates to the plasma membrane and is linked to CD44 and the actin cytoskeleton, which leads to increased cell motility and invasion [72].

During acute infection, gammaherpesviruses have been reported to induce long, branched cell projections, resembling the US3-mediated phenotype in alphaherpesvirus infections described above. Like the alphaherpesvirus US3-induced cell projections, gammaherpesvirus-induced membrane protrusions contain actin and appear to promote intercellular virus spread [73,74]. Despite this remarkable similarity in phenotype between alpha- and gammaherpesviruses, the underlying mechanism is different since US3 is conserved in alphaherpesviruses, but not in beta- or gammaherpesviruses. Cell projections have been described for the human gammaherpesvirus EBV and the murine gammaherpesvirus MHV-68. In both cases, the trigger for cell projection formation has been identified as the viral integral membrane protein complex consisting of BDLF2/BMRF2 for EBV and its ortholog gp48/ORF58 in MHV-68 [73,74]. This correlates with the observation that these protein complexes are involved in gammaherpesvirus cell-to-cell spread [75,76]. How these membrane protein complexes activate cell projection formation is unclear but experiments with RhoA dominant active/negative constructs demonstrated that the process critically relies on Rho GTPase signaling [73,74].

## 4. Conclusions

Increasing evidence points at the importance of cytoskeletal signaling in several key phases of herpesvirus infection, including entry, lytic infection and latent infection. However, many questions remain for future research in this relatively young field of research. Herpesviruses may embark on different uptake routes, depending on the virus and the host cell type. Recent data suggest that herpesvirus entry may be generally associated with Rho GTPase signaling, although the specifics and consequences are different for different herpesviruses and different host cells. It will therefore be interesting to further define the impact of the nature of Rho GTPase signaling on the specific entry pathway employed by herpesviruses in different cell types, and *vice versa*. A conserved feature of many, if not all, herpesvirus subfamilies seems to be the role of cytoskeletal signaling in the efficient delivery of virions to the nucleus. PI3K signaling appears to play a central role during this stage of viral infection. The underlying mechanism is not clear, although it may involve increased dynein-mediated transport through acetylation of microtubules [36,50].

Most of the studies on Rho GTPase signaling during viral entry have focused on signaling initiated by binding of envelope proteins with cell surface receptors. However, herpesvirus access to the cytoplasm involves release of tegument proteins in the cytosol. The potential impact of these tegument proteins on signaling pathways, including Rho GTPase pathways, is largely unknown but may be biologically relevant. The fact that at least one alphaherpesvirus tegument protein, US3, has been reported to interfere with Rho GTPase signaling [61] may be in line with this hypothesis.

During acute infection, severe rearrangements of the actin cytoskeleton including actin stress fiber disassembly and the formation of cell projections have been reported for alpha- and gammaherpesviruses. It is not surprising that Rho GTPase signaling plays a critical role in these actin rearrangements, but the underlying mechanisms are not fully understood. Although the role of US3 in the actin reorganization appears to be well conserved in alphaherpesviruses, it remains to be determined whether US3 acts through one conserved mechanism or whether different alphaherpesvirus US3 orthologs employ different mechanisms to manipulate actin. Although gammaherpesviruses induce similar actin rearrangments as observed for alphaherpesviruses, the viral triggers are different. It is unclear at present whether the lack of reports on similar phenotypes in betaherpesviruses indicates that these types of actin rearrangments and viral spread do not occur in this herpesvirus subfamily or whether this has not been investigated specifically as of yet. In this context, it is interesting that viral proteins from evolutionary much more distinct virus families induce similar actin rearrangements, including F11L of vaccinia virus and Nef of HIV [77–79]. Further unraveling the mechanistic details of interference of herpesviruses with Rho GTPase signaling may therefore reveal common threads that deserve further attention as potential antiviral drug targets.
